# miR-4732-3p in Extracellular Vesicles From Mesenchymal Stromal Cells Is Cardioprotective During Myocardial Ischemia

**DOI:** 10.3389/fcell.2021.734143

**Published:** 2021-08-31

**Authors:** Rafael Sánchez-Sánchez, Marta Gómez-Ferrer, Ignacio Reinal, Marc Buigues, Estela Villanueva-Bádenas, Imelda Ontoria-Oviedo, Amparo Hernándiz, Hernán González-King, Esteban Peiró-Molina, Akaitz Dorronsoro, Pilar Sepúlveda

**Affiliations:** Regenerative Medicine and Heart Transplantation Unit, Instituto de Investigación Sanitaria La Fe, Valencia, Spain

**Keywords:** extracellular vesicles, mesenchymal stromal (stem) cell (MSC), miR-4732-3p, angiogenesis, myocardial infarction, fibrosis, cardiac function analysis

## Abstract

Extracellular vesicles (EVs) derived from mesenchymal stromal cells (MSCs) are an emerging alternative to cell-based therapies to treat many diseases. However, the complexity of producing homogeneous populations of EVs in sufficient amount hampers their clinical use. To address these limitations, we immortalized dental pulp-derived MSC using a human telomerase lentiviral vector and investigated the cardioprotective potential of a hypoxia-regulated EV-derived cargo microRNA, miR-4732-3p. We tested the compared the capacity of a synthetic miR-4732-3p mimic with EVs to confer protection to cardiomyocytes, fibroblasts and endothelial cells against oxygen-glucose deprivation (OGD). Results showed that OGD-induced cardiomyocytes treated with either EVs or miR-4732-3p showed prolonged spontaneous beating, lowered ROS levels, and less apoptosis. Transfection of the miR-4732-3p mimic was more effective than EVs in stimulating angiogenesis *in vitro* and *in vivo* and in reducing fibroblast differentiation upon transforming growth factor beta treatment. Finally, the miR-4732-3p mimic reduced scar tissue and preserved cardiac function when transplanted intramyocardially in infarcted nude rats. Overall, these results indicate that miR-4732-3p is regulated by hypoxia and exerts cardioprotective actions against ischemic insult, with potential application in cell-free-based therapeutic strategies.

## Introduction

Coronary artery disease remains the primary cause of death in emerging and developed countries. Its most severe manifestation, acute myocardial infarction (AMI), is characterized by the occlusion of a coronary artery, which interrupts blood supply and oxygen and can lead to sudden death (Shao et al., 2020). Cell therapy is gaining traction as a promising alternative to conventional therapies for many diseases, including cardiac tissue repair. In this setting, mesenchymal stromal cells (MSCs) have been investigated in-depth, and are believed to act largely in a paracrine manner (Gnecchi et al., 2005; Armiñán et al., 2010). More recently, there has been a shift from cell (MSC)-based therapies to cell-free therapies, based on the use of adult stem cell-derived small extracellular vesicles (EVs), which can recapitulate the therapeutic benefits of parental cells (Rani et al., 2015). A particular strength of this approach is that EV-based therapies have fewer safety issues than cell-based therapies, and handling of these biological products is less complex (Zhang et al., 2021).

Extracellular vesicles are small particles 30–200 nm in diameter delimited by a lipid bilayer that are released from almost every type of cell. EVs are involved in intracellular communication, and transfer information via a diverse cargo of cytokines, membrane-trafficking molecules, chemokines, heat shock proteins and even mRNAs and microRNAs, which trigger myriad responses in target (recipient) cells including cardiac cells (Wu et al., 2020). In the setting of cardiac disease, EVs secreted by MSCs from different tissue origins have been demonstrated to mitigate ischemia/reperfusion (I/R) injury in different experimental models of AMI and I/R (Lai et al., 2010; Bian et al., 2014; Takov et al., 2020; Wu et al., 2020; Zhang et al., 2020).

The cardioprotective effects of MSC-derived EVs are due, at least in part, to different microRNA (miRNA) cargo molecules, which can modulate gene expression in recipient cardiac cells through post-transcriptional mRNA repression. The role of some of these miRNAs have been investigated in pre-clinical models. For instance, intramyocardial infusion of bone marrow-derived EVs overexpressing miR-486-5p from non-human primates was demonstrated to recover cardiac function after myocardial infarction in murine and non-human primates (Li et al., 2021). Similarly, delivery of MSC-derived EVs containing miR-150-5p attenuated adverse myocardial remodeling in a rat model of I/R (Ou et al., 2020). Likewise, MSC-EV-derived miR-21a-5p induced cardioprotection in mice after I/R injury (Luther et al., 2018), and adenovirus delivery of miR-148a prevented ventricular remodeling in mice subjected to pressure-overload (Raso et al., 2019). Mechanistically, miR-210 was found to enhance myocardial vascularization in a rat model of AMI after myocardial transduction by upregulating the expression of hepatocyte growth factor (Fan et al., 2018). Nonetheless, some cargo miRNAs from MSC-EVs can be deleterious to cardiac function, and strategies to reduce or eliminate specific packaged miRNA molecules have been shown to improve the anti-apoptotic and pro-angiogenic functionality of EVs (Zhang et al., 2017; Ning et al., 2021). In this scenario, it would be highly desirable to identify the optimal EV cargo and to design novel “miRNA cocktails” with selected miRNAs for specific applications such as cardioprotection (Jayawardena et al., 2014). With this aim, we sought to identify some key components that contribute to the therapeutic activity of MSC-derived EVs by evaluating several miRNAs contained in EV cargo. Here, we demonstrate that miR-4732-3p contributes to the therapeutic potential of EVs and induces protective mechanisms both in cardiomyocytes and endothelial cells. Furthermore, a miR-4732-3p mimic induced angiogenesis subcutaneously when implanted in a basement membrane plug and exerted cardioprotection when transplanted intramyocardially in a rat model of permanent coronary occlusion. These findings underscore miR-4732-3p as a potentially important player in ischemia-related processes with utility in cardioprotective strategies.

## Materials and Methods

### Ethics Statement

Animal procedures were approved by the institutional ethical and animal care committees according to guidelines from Directive 2010/63/EU of the European Parliament on the protection of animals used for scientific purposes, enforced in Spanish law under Real Decreto 1201/2005 (GVA authorized procedures 2020/VSC/PEA/0123 for the model of angiogenesis in NOD/SCID mice and 2020/VSC/PEA/0122 for the experimental model of myocardial infarction in nude rats).

### Primary Cultures

Neonatal rat cardiomyocytes (NRCM) were isolated as described (Armiñán et al., 2009) with some modifications. Briefly, rats aged 1 to 2 days were euthanized by decapitation, and the hearts were extracted, minced and incubated overnight in HBSS (without Ca and Mg) with 0.0125% trypsin (Sigma Aldrich) with gentle agitation at 4°C. Subsequently, the fragments were digested for 20 min at 37°C in an enzyme mixture (0.2% collagenase, 1 mg/mL DNAse) in Leibovitz L15 medium (all from Gibco-Invitrogen, Grand Island, NY, United States). Cell suspensions were collected and centrifuged at 90 × *g* for 5 min at room temperature and the cells were resuspended in Dulbecco’s Modified Eagle’s Medium (DMEM) containing 10% horse serum, 20% M-199 Medium, 5% fetal bovine serum (FBS) and 1% penicillin/streptomycin (all from Gibco). The cells were then incubated for 1.5 h under standard culture conditions (37°C in 5% CO_2_) to allow fibroblasts to attach to the culture plates. Then, the medium containing unattached cells was centrifugated and cardiomyocytes were pelleted and seeded in 0.1% gelatin-coated plates with DMEM, 4% horse serum, 17% M-199 Medium, 5% FBS, and 1% penicillin/streptomycin. Primary cultures of human umbilical vein endothelial cells (HUVEC; ATCC, Manassas, VA, United States) were grown in Vascular Cell Basal Medium supplemented with Endothelial Cell Growth Kit-VEGF (ATCC).

### EV Isolation

The immortal MSC-TERT line was used as previously described (Gómez-Ferrer et al., 2021). Donor cells were cultured in DMEM-low glucose supplemented with 1% penicillin/streptomycin and 10% FBS (Lonza, Basel, Switzerland). FBS was first ultracentrifuged at 100,000 *g* for 16 h to remove contaminant bovine EVs. Cells were incubated in a humidified atmosphere (37°C in 5% CO_2_) with EVs-free serum. EVs were collected after 48 h using a serial ultracentrifugation protocol (Gonzalez-King et al., 2017). Briefly, supernatants were first centrifuged at 2,000*g* for 10 min, then at 10,000*g* for 30 min and filtered through a 0.22-μmm filter. EVs were pelleted by ultracentrifugation at 100,000*g* for 70 min at 4°C (Optima L-100 XP, Beckman Coulter, Pasadena, CA, United States), washed with PBS, and pelleted again as before. Pellets were resuspended in 100 μl of PBS. Isolated EVs (10 mμL) were used for protein quantification with the BCA Protein Assay Kit (Pierce, Thermo Scientific Inc., Rockford, IL, United States). Where indicated, cells were treated with EVs by direct addition to the culture medium to a final concentration of 30 μg/mL shortly before the initiation of the different experimental conditions.

### EV Characterization

#### Western Blotting

Extracellular vesicles were suspended in RIPA buffer (1% NP40, 0.5% deoxycholate, 0.1% sodium dodecyl sulfate in Tris-buffered saline [TBS]) (Sigma-Aldrich, Madrid, Spain) supplemented with the protease inhibitors PMSF and leupeptin (Roche, Basel, Switzerland). Lysis was completed by five freeze-thaw cycles, followed by centrifugation at 12,000 *g* for 10 min at 4°C. EV protein concentration was determined as above. Equal amounts of samples were mixed with non-reducing Laemmli sample buffer (Bio-Rad, Hercules, CA, United States) and denatured at 96°C for 5 min. Proteins were separated on 10% SDS-polyacrylamide gels and transferred to polyvinylidene difluoride membranes (Immobilon-P; Millipore, Bedford, MA, United States). Membranes were blocked with TBS containing 5% (w/v) non-fat dry milk powder and 0.1% Tween-20. Human primary antibodies used for western blotting were as follows: anti-tubulin (dilution 1/4000; Sigma-Aldrich; T5168), anti-CD9 (Santa Cruz Biotechnology, Santa Cruz, CA, United States; C-4), anti-Tsg101 (dilution 1/200; Santa Cruz Biotechnology; C-2), anti-conexin43 (Cell Signaling Technology, Danvers, MA, United States) anti-ALIX (Santa Cruz Biotechnology), anti-HSP70 (Cell Signaling Technology), anti-cleavage-caspase 3 (Cell Signaling Technology), anti-SMA (Sigma-Aldrich) and anti-col I (Cell Signaling Technology). Detection was carried out using peroxidase-conjugated secondary antibodies and the ECL Plus Reagent (GE Healthcare, Little Chalfont, United Kingdom). Reactions were visualized using an Amersham Imager 600 (GE Healthcare) and quantified with ImageJ software (NIH).

#### Nanoparticle Tracking Analysis

Extracellular vesicle size distribution and quantification was performed using nanoparticle tracking analysis (NTA) on a NanoSight NS3000 System (Malvern Instruments, Malvern, United Kingdom). EV pellets were suspended in PBS in 200 μl of 0.22-μm-filtered PBS.

#### Electron Microscopy

Electron microscopy was performed as described (Garcia et al., 2016). Briefly, EVs were diluted in PBS, loaded onto Formwar carbon-coated grids, contrasted with 2% uranyl acetate and finally examined on a FEI Tecnai G2 Spirit transmission electron microscope. Images were acquired using a Morada CCD Camera (Olympus Soft Image Solutions GmbH, Münster, Germany).

#### EVs Small RNA Sequencing

Libraries were prepared using the SeqMatic TailorMix miRNA Sample Preparation Kit according to the manufacturer’s protocol. Briefly, 1 to 5 ng of RNA from EVs were subjected to adaptor 3’and 5’ ligation and first strand cDNA synthesis. Library amplification was performed by PCR using Indexed primers supplied in the kit. Final libraries were analyzed using Agilent Bioanalyzer to estimate the quantity and check size distribution and were sequenced on Illumina’s HiSeq2500.

### miRNA Electroporation of EVs

To incorporate miR-4732-3p into EVs, the later were resuspended in an electroporation buffer after the last ultracentrifugation step of isolation and were incubated with an artificial mimic of the chosen miRNA (MISSION miRNA mimic, Merck) at a concentration of 40 nM. The suspension was loaded into a Gene Pulser/MicroPulser Electroporation Cuvette and treated with 3 pulses of 300 V with 5 s between each pulse. Subsequently, the transduced EVs were incubated at 4°C for 30 min. To eliminate the miRNA not loaded in the EVs, the volume was increased with PBS to 25 mL and the solution was ultracentrifuged as described. The final pellet was resuspended in 100 μL of PBS.

### Generation of Lipofectamine miR-4732-3p Complexes

Transfection of miR-4732-3p mimic *in vitro* was assessed using Lipofectamine 2000 (Thermo Fisher Scientific) according to manufacturer’s instructions. Briefly, 100 μL of miR-4732-3p mimic 1 μg/μL was mixed with vigorous shaking for 30 s with 200 μL of Lipofectamine. The mixture was incubated for 30 min at room temperature.

### Encapsulation of miRNA-4732-3p Mimic

Transfection of miR-4732-3p mimic *in vivo* was assessed using Maxsuppressor *in vivo* RNA-LANCEr-II lipidic reagent (Cosmo Bio Co., Ltd., Japan) following manufacturer’s instructions. Briefly, 8.5 μl of semi-dry Maxsuppressor *in vivo* RNA-LANCEr-II was mixed with 16 μl of miR-4732-3p mimic in a total volume of 25 μl of PBS to a final dose of 25 μg/heart in each rat. The mixture was sonicated and used fresh for *in vivo* surgical procedures.

### qPCR Detection of miRNA Expression in Cell Cultures

Reverse transcription was performed with the miRCURY LNA Universal RT miRNA PCR Kit (Qiagen, Madrid, Spain). The miRNA was quantified using a Viia TM 7 Real System and the results were analyzed with QuantStudio Real-Time PCR Software (Applied Biosystems, Foster City, CA, United States).

### Oxygen/Glucose Deprivation (OGD) Procedure

Oxygen-glucose deprivation was induced on NRCM by culture with DMEM without glucose, glutamine, and phenol red (Thermo Fisher Scientific) in a chamber at 1.5% O_2_.

### Annexin V Staining

Neonatal rat cardiomyocytes were seeded at 2 × 10^5^ cells/cm^2^ in 1% gelatin-coated plates. After OGD, the cells were detached from the plastic with trypsin/EDTA, and were washed and resuspended in binding buffer with annexin-V-FITC and propidium iodide for 15 min. The percentage of annexin-positive cells was measured by flow cytometry in a FACS Canto II cytometer (BD Bioscience, San Jose, CA, United States).

### Lactate Dehydrogenase Assay

Neonatal rat cardiomyocytes were seeded at 2 × 10^5^ cells/cm^2^ in 1% gelatin-coated plates. After OGD, the supernatant was tested for lactate dehydrogenase using the Cytotoxicity Detection KitPLUS (LDH) (Roche, Indianapolis, IN, United States).

### Cell Rox Staining

Hypoxia-treated cells were washed three times with PBS and incubated with CellRox-FITC^®^ (Thermo Fisher) at a dilution of 1/200 in DMEM with no FBS. After 20 min incubation at 37°C the cells were washed three times in PBS to eliminate the unconjugated stain and were detached with trypsin/EDTA. The fluorescence intensity of the staining was measured by flow cytometry.

### NRCM Beating Measurement

To test the effect of OGD and EV treatment on cardiac contraction, we seeded cardiomyocytes at high confluence in gelatin-coated 6-well plates (5 × 10^5^ cells/well). After several days cardiomyocytes start to beat spontaneously. The number of beats per minute was measured using a Leica DM6000 inverted optical microscopy at 10 × magnification. Cells were treated with 30 μg/mL of EVs or transfected with 20 nM encapsulated miR-4732-3p using Lipofectamine as indicated above and were then subjected to OGD for 6 h, and each hour the beats were counted and normalized to non-treated cells.

### Cardiac Fibroblast Scratch Assay

Fibroblasts from neonatal rats were seeded in a 48-well plate at 2 × 10^5^ cells/well. Fibroblasts were treated the day before the analysis with 10 ng/mL of TGF-β to stimulate differentiation into myofibroblasts. Cells were then treated with 30 μg/mL of EVs or transfected with 20 nM miR-4732-3p using lipofectamine as indicated above. A straight line in the monolayer of fibroblast was created using a 20-μL pipette tip. Images were taken 24 h after the addition of treatments using a Leica DM600 inverted microscope at 4 × magnification. The area of the scratch wound was then measured using ImageJ.

### Immunofluorescence

Cardiac fibroblasts were cultured on glass coverslips at 2 × 10^4^ cell/glass in a 24-well plate. After treatment with TGFβ and EVs, cells were fixed in 4% paraformaldehyde for 10 min, washed three times with PBS and permeabilized with 0.1% Triton X-100 in PBS for 25 min. Cells were then washed with PBS and blocked with 10% FBS at room temperature for 30 min. Next, the cells were incubated with anti-actin, α-smooth muscle-Cy3 (Sigma-Aldrich) or anti-col1A (Cell Signaling Technology) at a concentration of 1/200 in a humidified incubator overnight. After washing in PBS with PBS, and in the case of col1A treated with the Alexa488 Donkey anti-rabbit (BD Bioscience) for 1 h, the cells were washed with PBS and counterstained with DAPI for 15 min and mounted using a drop of FluorSave (CalbioChem, San Diego, CA, United States). Images were captured with a Leica DM2500 fluorescence microscope.

### Tube Formation Assay

Tubular-like formations were measured using a described assay (Gonzalez-King et al., 2017). HUVEC cells were counted and seeded at 1.5 × 10^4^ cells/well in a 96-well plate pre-coated with 50 μL of growth factor-reduced Matrigel^®^ (BD Bioscience). Cells were incubated in a starvation medium to simulate the conditions of the infarcted heart (DMEM no glucose, 1% FBS) for 6 h with 30 μg/mL of EVs from MSC-TERT cells or transfected with 20 nM miR4732-3p. Images were taken using a Leica DM600 microscope at 10 × magnification and were analyzed using the online software Wimasis WimTube (WimTube: Tube Formation Assay Image Analysis Solution. Release 4.0^1^).

### Matrigel Plug Angiogenesis Assay

Angiogenesis was assayed *in vivo* by measuring blood vessel formation from subcutaneous tissue sprouted into a semi-solid gel of basement membrane containing the test sample, as described (Malinda, 2009). Growth factor-reduced Matrigel (350 μL) was mixed in liquid form at 4°C with 50 μL of EVs or 20 μg of encapsulated miRNA mimic (57 ng/μL). The Matrigel mix was injected subcutaneously into the flanks of 6–8-week-old athymic nude mice. After 14 days, mice were sacrificed and the Matrigel plugs were excised and fixed in 4% formaldehyde in PBS. Plugs were embedded in paraffin, sectioned, and immunostained with anti-CD31 antibodies and examined for growth of blood vessels. As a positive control, 400 μL of Matrigel containing 100 ng/mL fibroblast growth factor (FGF) and 20 U of heparin were injected, and as a negative control 400 μL of Matrigel containing saline vehicle were injected.

### Experimental Model of Myocardial Infarction

#### Animals

Nude rats weighing 200–250 g (HIH-Foxn1 rnu; Charles River Laboratories Inc., Wilmington, MA, United States) were used for infarction studies. The initial number of animals included in the study was 70. Mortality in all groups due to surgical procedures was ∼30%.

#### Myocardial Infarction and Cell Transplantation

Permanent ligation of the left coronary artery and intramyocardial transplantation was performed as described (Cerrada et al., 2013). Briefly, immediately after permanent left anterior descendent artery (LAD) ligation, rats were transplanted intramyocardially (saline, 3.5 × 10^10^ EVs or 25 μg miR-4732-3p mimic per animal) in two injections of 10 μL, at two discrete locations of the infarct border zone with a Hamilton syringe).

#### Functional Assessment by Echocardiography

Transthoracic echocardiography was performed in rats under inhalatory anesthesia (Sevorane) using an echocardiographic system (General Electrics, Milwaukee, WI, United States) equipped with a 10-MHz linear-array transducer, as previously reported (Gandia et al., 2008). Measurements were taken at baseline and post-transplantation (4 weeks). M-Mode and two-dimensional (2D) echocardiography was performed at the level of the papillary muscles in the parasternal short axis view. Functional parameters over five consecutive cardiac cycles were calculated using standard methods (Litwin et al., 1994). Left ventricular (LV) dimensions in end diastole (LVDd) and end systole (LVDs), anterior and posterior wall (AW and PW) thickness in diastole and systole, end-diastolic area (EDA) and end-systolic area (ESA) were measured. Fractional area change (FAC) was calculated as FAC = [(EDA–ESA)/EDA] × 100. Fractional Shortening (FS) was calculated as FS = [(LVDd-LVDs)/LVDd] × 100.

#### Measurements of Infarct Size

Left ventricle infarct size was measured in 8–12 transverse sections of 7 μm (1 slice each 200 μm of tissue) from apex to base in hearts fixed with 2% paraformaldehyde and stained with Masson’s trichrome. The fibrotic zone was determined by computer planimetry (Image-Pro Plus 7.1 software). Infarct size was expressed as percentage of total left ventricular area and as a mean of all slices from each heart.

### Statistical Analysis

Data are represented as mean ± SD. Student’s *t*-test was used for unpaired samples in the comparison between groups and the Chi-Square test was used when contingency tables were analyzed. When the distribution was not normal the Mann-Whitney *U* test was used. Analyses were conducted with GraphPad Prism 8 software. Differences were considered statistically significant at *p* < 0.05 with a 95% confidence interval.

## Results

### Identification of miR-4732-3p in Extracellular Vesicles

**FIGURE 1:**
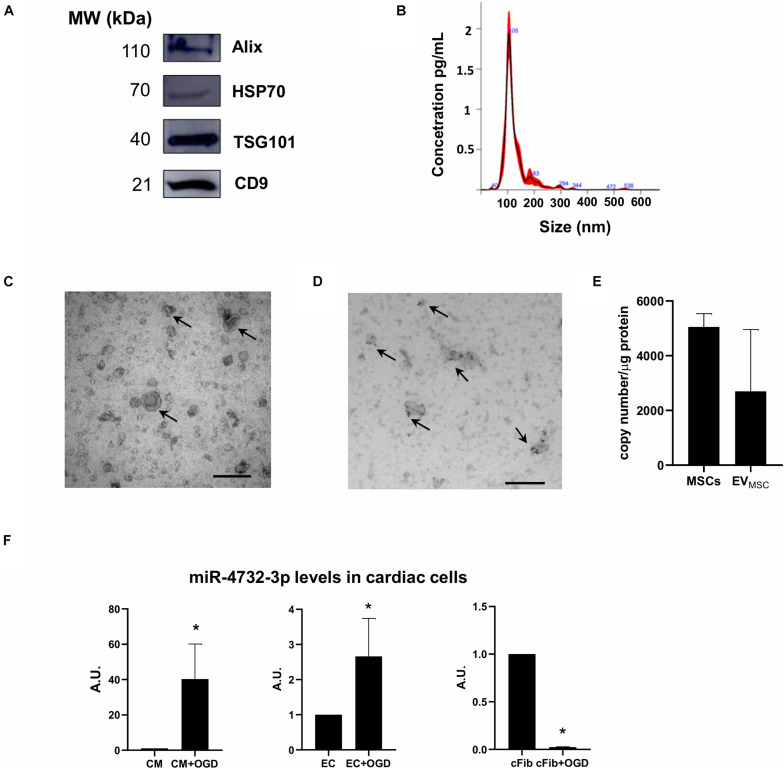
Characterization of EVs from immortalized dental pulp-derived MSC. **(A)** Representative western blot analysis of extracellular vesicle markers. **(B)** Representative histogram of EV size analyzed with the NanoSight NS300 instrument (*n* = 3). **(C)** Representatives image of isolated EVs analyzed by electron microscopy. **(D)** Representative image of EVs stained with immunogold anti-CD63 antibody and analyzed by electron microscopy. **(E)** Levels of miR-4732-3p in MSCs and in EVs released from these cells (*n* = 3). **(F)** Levels of miR-4732-3p in neonatal rat cardiomyocytes (NRCM) (*n* = 3), human umbilical cord blood vein cells (EC) (*n* = 4) and cardiac fibroblasts (cFib) (*n* = 4) in the presence or absence of oxygen glucose deprivation (OGD) (*p < 0.05). Scale bar = 200 nm.

Extracellular vesicles are postulated to be effective for cardiac repair (Zhao et al., 2015; Forsberg et al., 2020; Nazari-Shafti et al., 2020). However, the complexity involved in producing homogeneous populations in sufficient quantity hampers their clinical use. To overcome this problem, we previously immortalized dental pulp-derived primary MSC cultures by overexpressing human telomerase enzyme (termed MSC-T) using a lentiviral vector that provided resistance to hygromycin and increased telomerase activity. MSC-T exhibit prolonged lifespan and maintain cell division long after equivalent unmodified MSCs reach replicative senescence (Gómez-Ferrer et al., 2021). We harvested EVs from conditioned media of MSC-T cells using ultracentrifugation as described (Gonzalez-King et al., 2017). EVs from MSC-T were characterized by western blotting, nanotracking analysis and electron microscopy. The presence of the tetraspanin markers ALIX, HSP70, TSG101, and CD9 confirmed the presence of EVs (Figure 1A). The concentration and size of these vesicles was determined by nanotracking analysis and revealed structures of 100–130 nm (Figure 1B). This was confirmed by electron microscopy, which revealed circular membranous structures of 100 nm (Figure 1C), and further analysis of tetraspanins by immunogold staining with anti-CD63 antibodies revealed the accumulation of gold nanoparticles in vesicular structures (Figure 1D). We next analyzed the EV cargo by RNA-seq analysis and we examined for miRNAs that were regulated by hypoxia in parental MSC-T. Among them, we identified miR-4732-3p (Figure 1E), which was also found in cardiac cells at different intracellular abundance. Analysis of absolute copy number of miR-4732-3p per 0.5 × 10^6^ seeded cells was 73.46 ± 119.49 copies in NRCM 434.45 ± 134.51 in human umbilical vein endothelial cells (EC) and 595.02 ± 159.38 in rat cardiac fibroblasts (cFib). When these cultures were independently subjected to oxygen/glucose deprivation (OGD), the levels of intracellular miR-4732-3p were measured by qPCR and normalized to miRNA levels in cells cultured in standard conditions. These levels were significantly upregulated in NRCM and EC but downregulated in cFib (Figure 1F), indicating that this miRNA might play a specific role in different cardiac populations.

### miR-4732-3p Exerts Cardioprotective Effects on Cardiomyocytes Subjected to Oxygen and Glucose Deprivation

**FIGURE 2:**
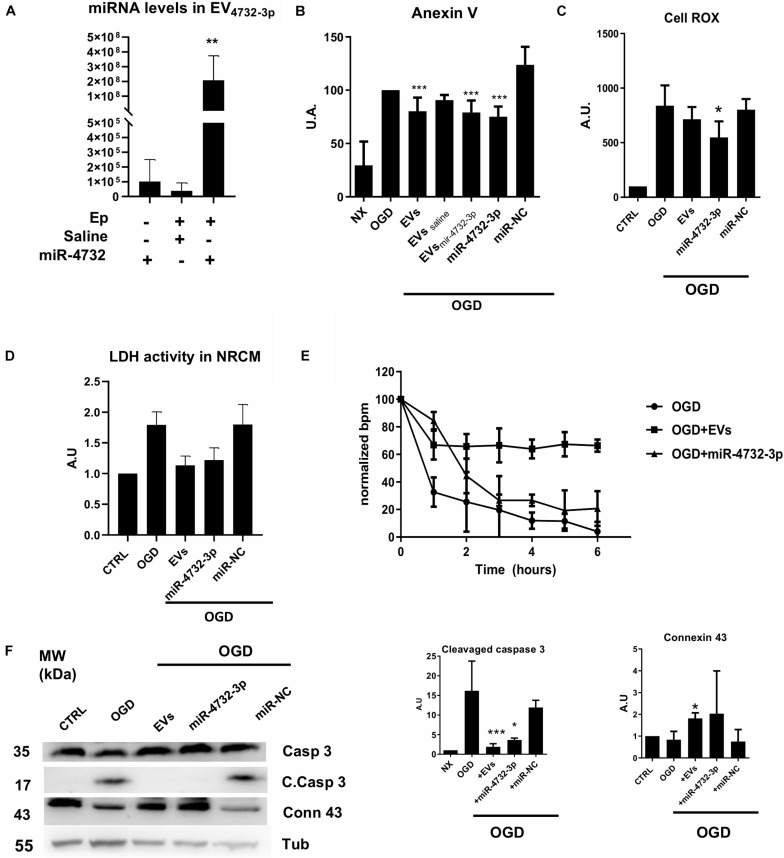
Effect of EVs and miR-4732-3p treatment in NRCM. **(A)** Quantification of miR-4732-3p levels in electroporated EVs (Ep = 3 pulses of 300V) with 40 nM miRNA or saline in the electroporation buffer (*n* = 3, *p < 0.05, **p < 0.01). **(B)** Effect of EVs on NRCM treated with oxygen and glucose deprivation (OGD) for 6 h. NRCM were treated with 30 μg/mL of EVs without electroporation (EVs), EVs electroporated with saline (EVs saline) or EVs electroporated with 20 nM miRNA hsa-4732-3p (EVs4732-3p). In addition OGM-treated NRCM were transfected with miR-4732-3p in max suppressor reagent (see section “Materials and Methods”) (miR-4732-3p) (*n* = 3, ***p < 0.001). **(C)** Quantification of ROS by Cell Rox kit measured in cardiomyocytes under OGD and treated with 30 μg/mL of EVs (EVs)transfected with 20 nM miR-4732-3p (miR-4732-3p) or 20 nM of miR-Negative control (miR-NC) (*n* = 3, *p < 0.05). **(D)** Relative quantification of lactate dehydrogenase activity (LDHA) measured by the absorbance in cardiomyocytes after OGD (*n* = 3). **(E)** Relative quantification of the contraction capacity of the cardiomyocytes *in vitro* during the OGD episode. **(F)** Representatives western blot images of cleavaged caspase 3 (C. Cas3) and connexin 43 (Conn 43) and α tubulin (Tub) (*n* = 3, *p < 0.05 and ***p < 0.001).

Extracellular vesicles are known to mediate cardioprotection in therapeutic interventions designed to mitigate I/R cardiac injury, and it is believed that EV miRNAs play a pivot role in this process (Barile et al., 2017; Zhang et al., 2017; Wu et al., 2020). To analyze the potential therapeutic effect of miR-4732-3p, we first electroporated EVs with a miR-4732-3p mimic to increase its concentration in EV particles (Figure 2A). Of note, electroporation did not affect the concentration or size of the EVs (Supplementary Figure 1) and they remained suitable for functional studies although the loss of miRNA cargo due to electroporation process is unknown. As an alternative to electroporation, synthetic miR-4732-3p was formulated into liposomes as a delivery system before transfection. The anti-apoptotic effect of EVs, with or without electroporated miRNA-4732-3p, and also miR-4732-3p liposome complexes, was assessed in NRCMs cultured under OGD conditions. We found that the number of apoptotic NRCMs was significantly reduced to a similar degree by the addition of native EVs or EVs previously electroporated with the miR-4732-3p mimic. Interestingly, a similar level of cardioprotection was achieved with liposomal miR-4732-3p (Figure 2B). Of note the control electroporation of EVs with saline reduced their anti-apoptosis action, likely due to the loss of vesicle integrity. In the context of electroporation, EVs that incorporated miRNA-4732-3p showed improved anti-apoptotic effects over electroporated vesicles with no miRNA (EVs_saline_) or scramble miRNA (not shown). The finding that transfected miR-4732-3p could induce a similar reduction in apoptosis to addition of EVs underlines the potential role of this miRNA in cardioprotective mechanisms.

### miR-4732-3p Recapitulates the Therapeutic Effects of EVs on OGD Injured Cardiac Cells

Analysis of reactive oxygen species production and LDH activity in NRCMs treated with either EVs or transfected with miR-4732-3p mimic before OGD revealed similar levels of cardioprotection (Figures 2C,D). While the amount of miR-472-3p delivered by EVs is much lower than the transfected amount, it is likely that other miRNAs in EVs play a role in the cardioprotection. To analyze the global response of miR-4732-3p in comparison with EVs, we tested the contractile profile of cardiomyocytes during ischemia. When we added EVs to NRCM cultures before the OGD episode, we observed that cells maintained contraction capacity in comparison with untreated NRCMs (Figure 2E). NRCMs transfected with miR-4732-3p also showed an improvement in their contraction capacity but the effect was not as pronounced (Figure 2E). To further explore this mechanism, we analyzed the levels of connexin 43 and caspase 3 by western blotting. Results showed that whereas cleaved caspase 3 was less abundant in NRCMs treated with EVs or miR-4732-3p suggesting a possible interaction of the miRNA with this pathway. However, further experiments are needed to confirm this interaction like a Cas3 inhibition assay. EVs exerted a greater upregulation of connexin 43, which could explain why EVs were more effective in preserving cell beating after cardiac injury (Figure 2F).

### miRNA-4732-3p Modulates the Migration Capacity of Fibroblasts

**FIGURE 3:**
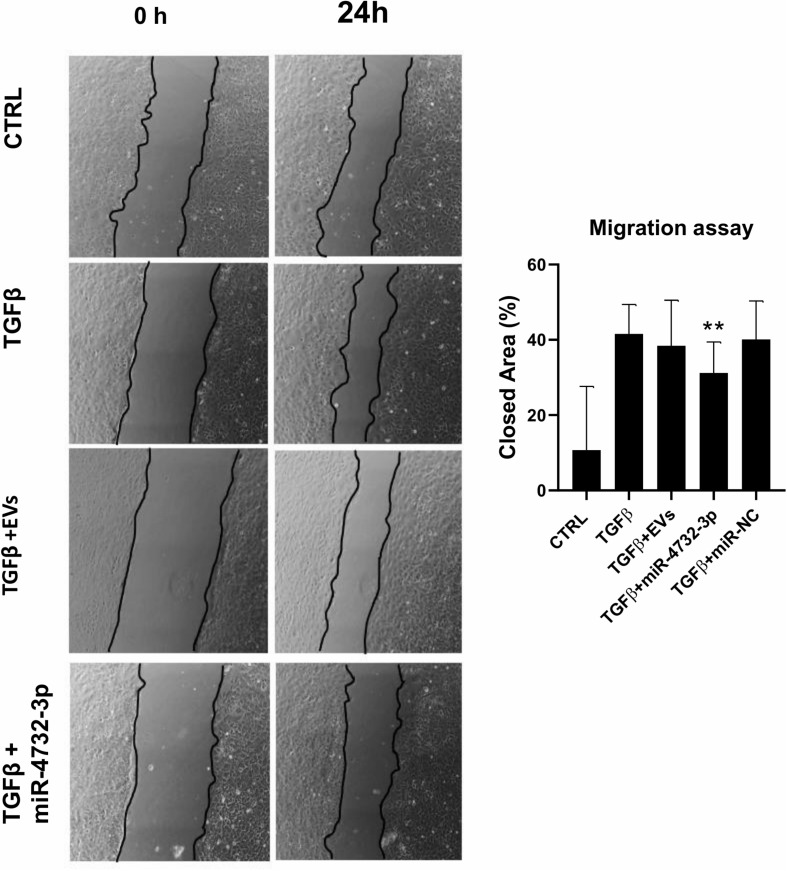
Effect of EVs and miRNA 4732-3p on fibroblast migration. The percentage of closed area was calculated from the difference between the area at baseline and after 24 h. Fibroblasts were cultured in standard culture condition (CTRL) or treated with 10 ng/mL of TGFβ alone or with 30 μg/mL of EVs (TGFβ + EVs) transfected with 20 nM of lipofectamine-miRNA 4732-3p complexes or 20 nM of miR-Negative Control. Images were captured on an inverted microscope at 4 × magnification. The area of the scratch was measured using ImageJ (*n* = 6, **p < 0.01).

We next sought to analyze the response of cardiac fibroblasts (cFib) to miR-4732-3p since these cells play an active role in myocardial remodeling after injury. Among the putative target genes of miR-4732-3p are FGFs, angiotensin II and members of the TGFβ family including TGFβ, TGFβR1/3 and SMAD2/4^2^ (not shown). To test the anti-fibrotic effect of miR-4732-3p, we added EVs or the miR-4732-3p mimic to fibroblasts treated with TGFβ using a scratch assay. Results confirmed that the migratory capacity was lower in fibroblasts transfected with miRNA-4732-3p and treated with TGFβ than in control TGFβ-treated fibroblasts. By contrast, EV treatment failed to affect the pro-migratory activity of TGFβ (Figure 3).

### miRNA 4732-3p Reduces the Expression of Myofibroblast Markers in Cardiac Fibroblasts

**FIGURE 4:**
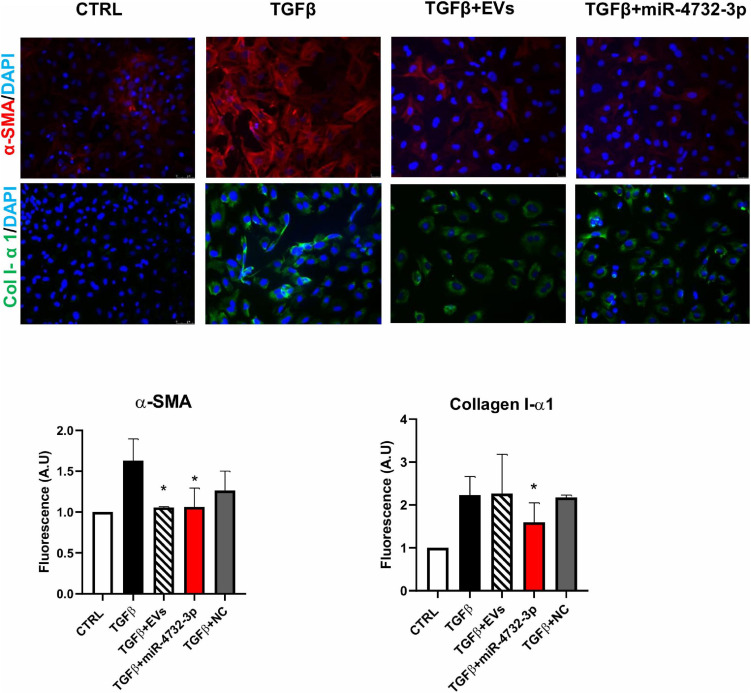
Effect of EVs and miRNA 4732-3p on the expression of the myofibroblast markers α-smooth muscle actin (α-SMA) and collagen 1 α (Col1). Fibroblasts were cultured in standard culture condition (CTRL) or treated with 10 ng/mL TGFβ (TGFβ),with or without 30 μg/mL of EVs (TGFβ + EVs), 20 nM of lipofectamine-miR 4732-3p complexes or 20 nM of miR-Negative Control. Images were taken with a fluorescence microscope and the fluorescence was quantified using ImageJ (*n* = 3, *p < 0.05).

A major consequence of ischemic damage is the formation of a scar in the infarcted area derived from the differentiation of fibroblasts to myofibroblasts (Shinde and Frangogiannis, 2014). After myocardial infarction, a cytokine storm induces myofibroblast differentiation via angiotensin II and TGFβ, among others (Frangogiannis, 2015), Myofibroblasts have a greater contractile and invasion capacity and also a higher production of extracellular matrix components; hence, the excessive differentiation to myofibroblasts results in scar formation with no contraction capacity. To evaluate the effect of miR-4732-3p on myofibroblast induction, we treated cardiac fibroblasts with 10 ng/mL TGFβ to simulate differentiation. Myofibroblast differentiation was characterized by an increase in the expression of smooth muscle actin (α-SMA) and increased production of extracellular matrix proteins like collagen IA (col I-α1) (Figure 4). Although both EVs and transfection of miR-4732-3p led to a reduction in α-SMA expression, only miR-4732-3p treatment reduced collagen production. Taken together, the results suggest that miR-4732-3p inhibits myofibroblast differentiation and the production of extracellular matrix, which might control fibrosis in the ischemic area.

### miR-4732-3p Has Angiogenic Effects in Endothelial Cells *in vitro* and *in vivo*

**FIGURE 5:**
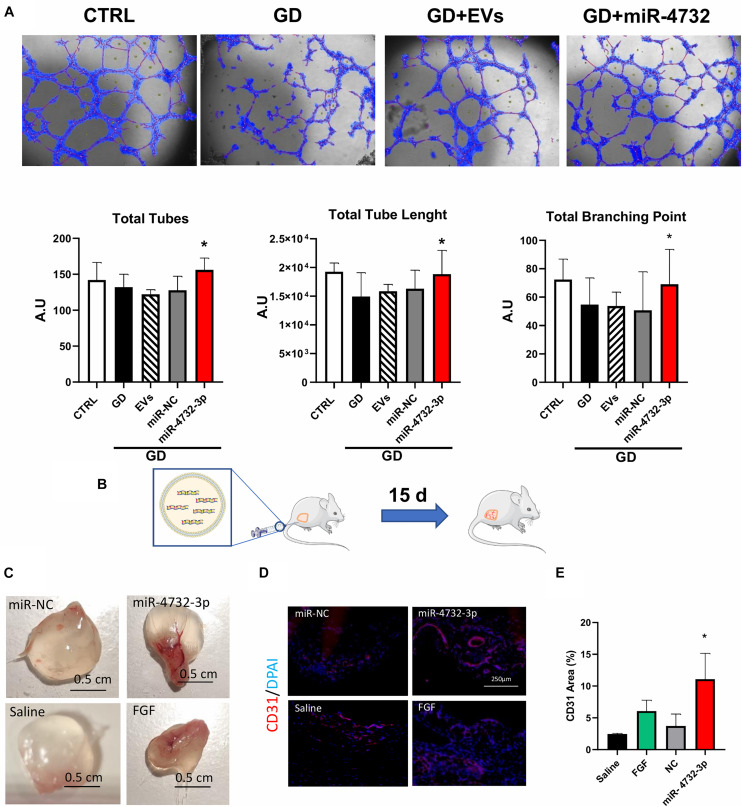
Effect of EVs and miRNA 4732-3p on angiogenesis. **(A)** Representative images and quantification of the tubular structures in endothelial cells in standard culture condition (CTRL) in glucose deprivation (GD), with or without 30 μg/mL of EVs(EVs), 20 nM lipofectamine-miRNA-4732-3p complexes (miR-4732-3p) or 20 nM of a negative control miRNA (miR-NC) and quantification of total tubes, total tube length and total branching points. **(B)** Scheme of Matrigel plug assay in nude mice to measure angiogenesis. Mice were divided into four groups. Matrigel was mixed with saline, 100 ng/mL FGF and 20 U heparin (FGF), 20 μg of negative control miRNA (miR-NC) or 20 μg of miR-4732-3p in Maxsuppressor reagent (miR-4732-3p). Mice were sacrificed 15 days after Matrigel injection. **(C)** Representatives images of extracted plugs (scale-bar = 0.5 cm). **(D)** Representative images of CD31-positive staining in the plugs by immunohistochemistry. **(E)** Quantification of CD31-positive area in the plugs (*n* = 5, *p < 0.05).

We tested the capacity of miR-4732-3p to induce the formation of new vasculature *in vitro*. We did not use OGD treatment since in those conditions tube formation was drastically impaired. Instead, HUVEC were seeded in DMEM without glucose and glutamine (Thermo Fisher Scientific) in growth factor-reduced Matrigel (GD condition). Results showed a significant increase in the formation of tubular structures from HUVEC transfected with the miR-4732-3p compared with untreated cells (Figure 5A). Contrastingly, cells treated with EVs showed no significant increase in vascular structures at the doses analyzed. We next sought to test the angiogenic potential of miR-4732-3p *in vivo*. Nude mice were injected subcutaneously with a Matrigel plug containing either saline, FGF (as a positive control), EVs or miR-4732-3p (Figure 5B). Plugs were removed 15 days later and were embedded in paraffin for histological analysis (Figure 5C). Individual pugs are shown in Supplementary Figure 2. Quantification of angiogenesis was performed by measuring plug-infiltrating vessels using anti-CD31 staining. The results showed a potent angiogenic effect in mice treated with miR-4732-3p in comparison to EVs and control conditions (Figures 5D,E). Overall, these findings indicate that miR-4732-3p induces angiogenesis both under stress and in physiological conditions.

### miRNA-4732-3p Improves Cardiac Function and Reduces Infarct Size

The ability of EVs derived from MSC isolated from different origins has been extensively reported and in most cases therapeutic effects have been attributed to miRNA cargo components (Wang et al., 2017; Luther et al., 2018; Zhu et al., 2018; Mao et al., 2019; Huang et al., 2020; Peng et al., 2020; Xu et al., 2020).

**FIGURE 6:**
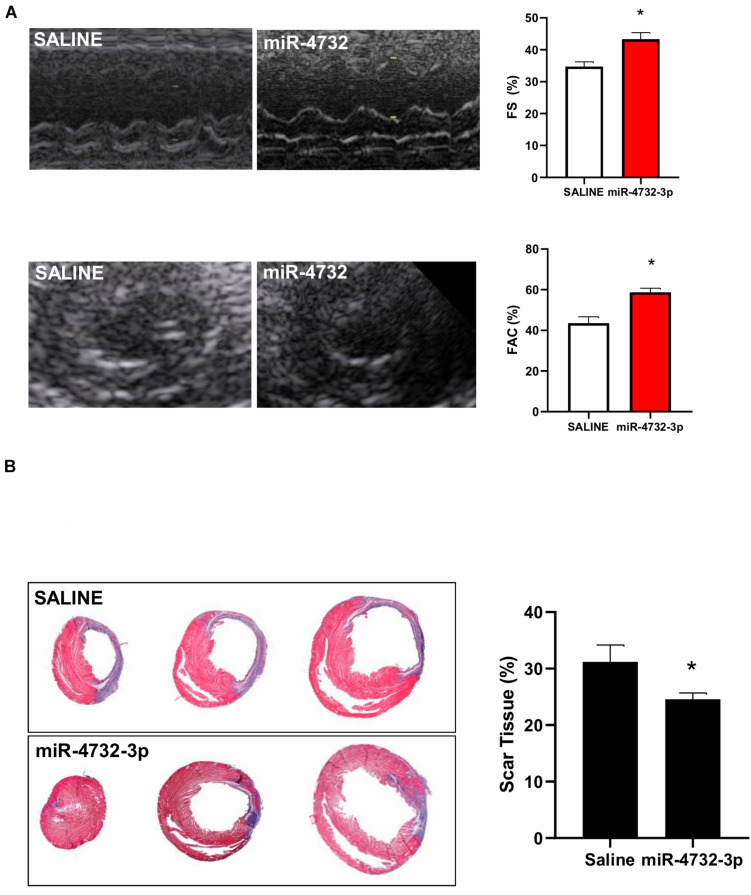
Effect of EVs and miRNA 4732-3p in infarcted nude rats **(A)** improvement of LV function in miR-4732-3p-treated animals. Representative echocardiographic images of M-mode and two-dimensional systolic frame showing differences in wall motion in one animal from saline (*n* = 6), and miR-4732-3p (*n* = 8) groups. Quantified values of fractional shortening (FS) area fractional change (FAC) are given. Data are expressed as mean ± SEM (*p < 0.05 in both panels). **(B)** Fibrotic area (blue color) in the LV was calculated from Masson’s Trichrome stained sections. Animals were euthanized 4 weeks post-transplantation. Values are the mean ± SEM (*p < 0.05).

We tested whether the administration of miR-4732-3p could recapitulate this effect by encapsulating it in liposomes before intramyocardial implantation shortly after permanent LAD ligation in nude rats. Results were compared to saline intramyocardial injections. Four weeks after transplantation, cardiac function parameters were measured to assess the degree of functional recovery. The miR-4732-3p group displayed a significant recovery of systolic function as calculated by the percentage of fractional shortening (FS) and fractional area change (FAC) (Figure 6A). Treatment with miR-4732-3p also significantly reduced the area of fibrous scar tissue (Figure 6B). These *in vivo* data support the hypothesis that miR-4732-3p can induce cardioprotection against cardiac ischemia.

## Discussion

The lack of effective regenerative therapies in the treatment of ischemia-related diseases compels the development of new approaches to improve clinical outcomes. Recently, the field of cardiovascular therapies have moved to cell-free-based therapies using EVs derived from MSC. There is evidence to suggest that MSC-EVs isolated from different tissues are able to promote myocardial repair through protection of ischemic cardiomyocytes and preservation of cardiac function (Teng et al., 2015; Chen et al., 2020; Mathew et al., 2020; Takov et al., 2020). These biological products are cardioprotective in preclinical models of myocardial infarction, and increasing evidence indicates that miRNAs are the main cargo components responsible for the therapeutic potential of EVs. Nonetheless, the heterogeneous composition of EVs is a major hurdle for their clinical use, and simpler biological products would be desirable to induce tissue repair. Another concern for the use of MSC-derived EVs in the clinical context is the potential involvement of deleterious processes mediated by specific miRNAs. For example, miR-130b-3p in MSC-derived EVs has been shown to promote lung cancer cell proliferation, migration and invasion (Guo et al., 2021) and exacerbates I/R injury after adoptive transfer in diabetic mice (Gan et al., 2020). With the aim of identifying miRNAs responsible (at least in part) for the beneficial effects attributed to EVs, we analyzed miRNA content in MSC derived EVs and investigated a miRNA with cardioprotective features when transplanted as a single molecule.

We provide evidence that miR-4732-3p is contained in EVs released from dental pulp-derived MSC (Figure 1E). We transfected miR-4732-3p *in vitro* and *in vivo* in liposomes that allowed delivery into cells and tissues and compared its therapeutic effect with that of MSC-EVs. EVs have been shown to reduce oxidative stress and apoptosis and to induce angiogenesis and cardioprotection against I/R injury mediated by specific miRNAs (Arslan et al., 2013; Zhao et al., 2019; Chen et al., 2020; Wen et al., 2020) and so we focused on those mechanisms in this study. We show that miR-4732-3p: (i) reduces apoptosis, ROS levels and LDH activity in NRCMs after OGD; (ii) prevents fibroblast migration and myofibroblast differentiation; (iii) induces angiogenesis both *in vitro* and *in vivo*; and (iv) confers cardioprotection when injected intramyocardially in infarcted nude rats as measured by functional and morphometric studies.

miR-4732-3p is also present in cardiac cells where it is modulated by oxygen in a cell-specific manner. Hypoxia upregulates miR-4732-3p in NRCM and EC, which is in accordance with the cardioprotective responses induced by miR-4732-3p in these cell populations after transfection. However, a decrease in oxygen levels induces downregulation of miR-4732-3p in cFib. Accordingly, transfection of TGF-β-treated cFib with miR-4732-3p reduced their migration capacity and differentiation into myofibroblasts. Interestingly, miR-4732-3p targets the SMAD2 and SMAD4 components of TGF-β pathway (Doss et al., 2015), and so the hypoxia-induced downregulation of miR-4732-3p in response to OGD might translate to an upregulation of the TGF-β pathway, leading to cFib proliferation and myofibroblast differentiation.

Although the doses are not comparable, the paracrine effects observed after miR-4732-3p transfection were more robust than those exerted by EVs. In this context, miRNA therapy offers the advantage of a homogeneous content versus the heterogeneous content of vesicles, which would simplify scale-up of biological products. EVs electroporated in saline solution with no added miRNA did not offer cardioprotection of cultured cells, indicating the importance of the EV cargo. Our data do not dismiss the possibility that other miRNAs, metabolites or proteins from EVs could also induce strong cardioprotective effects but, as mentioned, the complexity of EV cargo together with intra-donor variability limits their exploitation in a clinical scenario. *In vivo* transfer of single miRNAs would overcome this problem. In this context, other studies have reported therapeutic effects of individual miRNAs present in EVs from MSC and other adult progenitor cells. As an example, a combination of miR-1, miR-133, miR-208, and miR-499 induced cardiac reprogramming in the infarcted heart and improved cardiac function in mice subjected to cardiac injury by permanent LAD artery ligation (Jayawardena et al., 2014), and adoptive transfer of miR-181b mimic recapitulated cardioprotection induced by exosomes derived from cardiosphere-derived cells (CDCs) in a rat model of MI (De Couto et al., 2017).

Because hypoxia preconditioning promotes the release of vesicles with enhanced therapeutic potential (Bister et al., 2020), we were interested in those miRNA present in EVs and modulated by hypoxia (unpublished results). miR-4732-3p is regulated by hypoxia in MSC, CM, EC, and cFib. We do not know if this miRNA is directly regulated by Hypoxia Inducible Factor 1 subunit α; however, HIF-1α inhibitor (HIF1AN) is a putative target gene of this miRNA, as assessesed by Targetscan^3^, Diana tools^4^, and miRDB^5^. miR-4732-3p upregulation by hypoxia could presumably downregulate HIF1AN, leading to stabilization of HIF-1α, as has been reported for other miRNAs including miR-335, which also confers cardioprotective effects (Wu et al., 2018).

The present study has some limitations. First, the therapeutic contribution of miR-4732-3p in comparison with other miRNAs present in EVs is unknown. The absolute copy number of this miRNA is relatively low in MSC-derived EVs so it is plausible that other miRNAs with higher abundance like miR-21a-5p (Luther et al., 2018), miR-26a (Park et al., 2018), miR-181a (Zilun et al., 2019), miR-210 (Wang et al., 2017), or miR-486-5p (Li et al., 2021) have a greater contribution (Nazari-Shafti et al., 2020). Second, no target genes have been validated for miR-4732-3p, but this molecule has been described as a key modulator of TGFβ signaling pathway in human erythropoiesis (Doss et al., 2015). The results presented here point in the same direction and indicate that the TGFβ pathway has a major contribution. Third, we did not perform loss-of-function experiments to analyze the effect of miR-4732-3p inhibition on myocardial repair. We previously observed low levels of miR-4732-3p in the serum of patients that experienced cardiotoxicity after doxorubicin challenge (Hervás et al., 2018). In addition to TGFβ, bioinformatic analysis identified components of Wnt/β-catenin pathways among the putative target genes of miR-4732-3p, supporting the idea that this molecule is cardioprotective and could be involved in cardiac turnover in the adult heart. In this context, in a study of monozygotic twins discordant for congenital heart disease, miR-4732-3p was among the most upregulated miRNAs in those with congenital heart disease, further linking this molecule to heart developmental processes (Abu-Halima et al., 2019).

In conclusion, and in agreement with previous reports, EVs derived from MSC have cardioprotective mechanisms in cardiac cells. We found that miR-4732-3p is present in EVs from MSC and can trigger angiogenic and cardioprotective responses compatible with the effect of EV therapy. When transplanted in murine models of angiogenesis and myocardial infarction, miR-4732-3p induced blood vessel formation, reduced scar formation and prevented cardiac function deterioration indicating that a single miR can recapitulate the therapeutic effects of EVs. These results support the potential paracrine actions of miR-4732-3p, but the mechanism by which this molecule operates in different cardiac cell populations requires further investigation. Additional experiments to identify 3’-untranslated regions in putative miR-4732-3p target genes are needed as a next step to identify the specific molecular pathways modulated by this miRNA.

## Data Availability Statement

The data presented in the study are deposited in the GEO database repository, accession number is GEO:GSE179940.

## Ethics Statement

The animal study was reviewed and approved by the Hospital La Fe Ethics and Animal Care Committee.

## Author Contributions

RS-S, MG-F, IR, MB, EV-B, HG-K, and IO-O were responsible for design, performance, and analysis and interpretation of the *in vitro* and *in vivo* experiments. EP-M and AH were responsible for cardiac function *in vivo* studies. AD was responsible for conceptualization, investigation, data curation, and review. PS was responsible for conceptualization, data curation, funding acquisition, writing, and review. All authors have read and agreed to the published version of the manuscript.

## Conflict of Interest

The authors declare that the research was conducted in the absence of any commercial or financial relationships that could be construed as a potential conflict of interest.

## Publisher’s Note

All claims expressed in this article are solely those of the authors and do not necessarily represent those of their affiliated organizations, or those of the publisher, the editors and the reviewers. Any product that may be evaluated in this article, or claim that may be made by its manufacturer, is not guaranteed or endorsed by the publisher.

## Footnotes

1
https://www.wimasis.com/en/WimTube

2
http://www.targetscan.org/vert_72/

3
http://targetscan.org

4
http://diana.imis.athena-innovation.gr

5
http://mirdb.org

## References

[B1] Abu-Halima M. Meese E. Saleh M. A. Keller A. Abdul-Khaliq H. Raedle-Hurst T. (2019). Micro-RNA 150-5p predicts overt heart failure in patients with univentricular hearts. *PLoS One* 14:e0223606. 10.1371/journal.pone.0223606 31600281 PMC6786722

[B2] Armiñán A. Gandía C. Bartual M. García-Verdugo J. M. Lledó E. Mirabet V. (2009). Cardiac differentiation is driven by nkx2.5 and gata4 nuclear translocation in tissue-specific mesenchymal stem cells. *Stem Cells Dev.* 18 907–918. 10.1089/scd.2008.0292 18983250

[B3] Armiñán A. Gandía C. García-Verdugo J. M. Lledó E. Trigueros C. Ruiz-Saurí A. (2010). Mesenchymal stem cells provide better results than hematopoietic precursors for the treatment of myocardial infarction. *J. Am. Coll. Cardiol.* 55 2244–2253. 10.1016/j.jacc.2009.08.092 20466205

[B4] Arslan F. Lai R. C. Smeets M. B. Akeroyd L. Choo A. Aguor E. N. E. (2013). Mesenchymal stem cell-derived exosomes increase ATP levels, decrease oxidative stress and activate PI3K/Akt pathway to enhance myocardial viability and prevent adverse remodeling after myocardial ischemia/reperfusion injury. *Stem Cell Res.* 10 301–312. 10.1016/j.scr.2013.01.002 23399448

[B5] Barile L. Moccetti T. Marbï£¡n E. Vassalli G. (2017). Roles of exosomes in cardioprotection. *Eur. Heart J.* 38, 1372–1379. 10.1093/eurheartj/ehw304 27443883

[B6] Bian S. Zhang L. Duan L. Wang X. Min Y. Yu H. (2014). Extracellular vesicles derived from human bone marrow mesenchymal stem cells promote angiogenesis in a rat myocardial infarction model. *J. Mol. Med.* 92, 387–397. 10.1007/s00109-013-1110-5 24337504

[B7] Bister N. Pistono C. Huremagic B. Jolkkonen J. Giugno R. Malm T. (2020). Hypoxia and extracellular vesicles: a review on methods, vesicular cargo and functions. *J Extracell. Vesicles* 10:e12002. 10.1002/jev2.12002 33304471 PMC7710128

[B8] Cerrada I. Ruiz-Saurí A. Carrero R. Trigueros C. Dorronsoro A. Sanchez-Puelles J. M. (2013). Hypoxia-inducible factor 1 alpha contributes to cardiac healing in mesenchymal stem cells-mediated cardiac repair. *Stem Cells Dev.* 22 501–511. 10.1089/scd.2012.0340 22873764

[B9] Chen Q. Liu Y. Ding X. Li Q. Qiu F. Wang M. (2020). Bone marrow mesenchymal stem cell-secreted exosomes carrying microRNA-125b protect against myocardial ischemia reperfusion injury via targeting SIRT7. *Mol. Cell. Biochem.* 465 103–114. 10.1007/s11010-019-03671-z 31858380 PMC6955239

[B10] De Couto G. Gallet R. Cambier L. Jaghatspanyan E. Makkar N. Dawkins J. F. (2017). Exosomal microRNA transfer into macrophages mediates cellular postconditioning. *Circulation* 136 200–214. 10.1161/CIRCULATIONAHA.116.024590 28411247 PMC5505791

[B11] Doss J. F. Corcoran D. L. Jima D. D. Telen M. J. Dave S. S. Chi J. T. (2015). A comprehensive joint analysis of the long and short RNA transcriptomes of human erythrocytes. *BMC Genomics* 16:952. 10.1186/s12864-015-2156-2 26573221 PMC4647483

[B12] Fan Z. G. Qu X. L. Chu P. Gao Y. L. Gao X. F. Chen S. L. (2018). MicroRNA-210 promotes angiogenesis in acute myocardial infarction. *Mol. Med. Rep.* 17:8620. 10.3892/mmr.2018.8620 29484401 PMC5866007

[B13] Forsberg M. H. Kink J. A. Hematti P. Capitini C. M. (2020). Mesenchymal stromal cells and exosomes: progress and challenges. *Front. Cell Dev. Biol.* 8:665. 10.3389/fcell.2020.00665 32766255 PMC7379234

[B14] Frangogiannis N. G. (2015). Pathophysiology of myocardial infarction. *Compr. Physiol.* 5 1841–1875. 10.1002/cphy.c150006 26426469

[B15] Gan L. Xie D. Liu J. Bond Lau W. Christopher T. A. Lopez B. (2020). Small extracellular microvesicles mediated pathological communications between dysfunctional adipocytes and cardiomyocytes as a novel mechanism exacerbating ischemia/reperfusion injury in diabetic mice. *Circulation* 141 968–983. 10.1161/CIRCULATIONAHA.119.042640 31918577 PMC7093230

[B16] Gandia C. Armiñan A. N. A. García-Verdugo J. M. Lledó E. Ruiz A. Miñana M. D. (2008). Human dental pulp stem cells improve left ventricular function, induce angiogenesis, and reduce infarct size in rats with acute myocardial infarction. *Stem Cells* 26 638–645. 10.1634/stemcells.2007-0484 18079433

[B17] Garcia N. A. Moncayo-Arlandi J. Sepulveda P. Diez-Juan A. (2016). Cardiomyocyte exosomes regulate glycolytic flux in endothelium by direct transfer of GLUT transporters and glycolytic enzymes. *Cardiovasc. Res.* 109, 397–408. 10.1093/cvr/cvv260 26609058

[B18] Gnecchi M. He H. Liang O. D. Melo L. G. Morello F. Mu H. (2005). Paracrine action accounts for marked protection of ischemic heart by Akt-modified mesenchymal stem cells. *Nat. Med.* 11 367–368. 10.1038/nm0405-367 15812508

[B19] Gómez-Ferrer M. Villanueva-Badenas E. Sánchez-Sánchez R. Sánchez-López C. M. Baquero M. C. Sepúlveda P. (2021). Hif-1α and pro-inflammatory signaling improves the immunomodulatory activity of MSC-derived extracellular vesicles. *Int. J. Mol. Sci.* 22:3416. 10.3390/ij22073416 PMC8036951 33810359

[B20] Gonzalez-King H. García N. A. Ontoria-Oviedo I. Ciria M. Montero J. A. Sepúlveda P. (2017). Hypoxia inducible factor-1α potentiates jagged 1-mediated angiogenesis by mesenchymal stem cell-derived exosomes. *Stem Cells* 35 1747–1759. 10.1002/stem.2618 28376567

[B21] Guo Q. Yan J. Song T. Zhong C. Kuang J. Mo Y. (2021). microRNA-130b-3p contained in MSC-derived EVs promotes lung cancer progression by regulating the FOXO3/NFE2L2/TXNRD1 axis. *Mol. Ther. Oncolytics* 20 132–146. 10.1016/j.omto.2020.09.005 33575477 PMC7851484

[B22] Hervás D. Garcia N. Santaballa A. Salvador C. Sánchez R. Panadero J. (2018). *EP18248213.3. Method for Predicting Cardiotoxicity Risk in Cancer Patients Receiving Anthracyclines Chemotherapy. Spain. 12/2018. Publication Number: WO/2020/136032. International Application No.PCT/EP2019/085388.* Münich: EPO.

[B23] Huang L. Yang L. Ding Y. Jiang X. Xia Z. You Z. (2020). Human umbilical cord mesenchymal stem cells-derived exosomes transfers microRNA-19a to protect cardiomyocytes from acute myocardial infarction by targeting SOX6. *Cell Cycle* 19 339–353. 10.1080/15384101.2019.1711305 31924121 PMC7028160

[B24] Jayawardena T. M. Finch E. A. Zhang L. Zhang H. Hodgkinson C. P. Pratt R. E. (2014). MicroRNA induced cardiac reprogramming in vivo: evidence for mature cardiac myocytes and improved cardiac function. *Circ. Res.* 116 418–424. 10.1161/CIRCRESAHA.116.304510 25351576 PMC4312531

[B25] Lai R. C. Arslan F. Lee M. M. Sze N. S. K. Choo A. Chen T. S. (2010). Exosome secreted by MSC reduces myocardial ischemia/reperfusion injury. *Stem Cell Res.* 4, 214–222. 10.1016/j.scr.2009.12.003 20138817

[B26] Li Q. Xu Y. Lv K. Wang Y. Zhong Z. Xiao C. (2021). *Small Extracellular Vesicles Containing miR-486-5p Promote Angiogenesis After Myocardial Infarction in Mice and Nonhuman Primates.* Available online at: http://stm.sciencemag.org/ (accessed March 18, 2021) 10.1126/scitranslmed.abb0202 33692129

[B27] Litwin S. E. Katz S. E. Morgan J. P. Douglas P. S. (1994). Serial echocardiographic assessment of left ventricular geometry and function after large myocardial infarction in the rat. *Circulation* 89 345–354. 10.1161/01.CIR.89.1.345 8281668

[B28] Luther K. M. Haar L. McGuinness M. Wang Y. Lynch T. L. IV Phan A. (2018). Exosomal miR-21a-5p mediates cardioprotection by mesenchymal stem cells. *J. Mol. Cell. Cardiol.* 119 125–137. 10.1016/j.yjmcc.2018.04.012 29698635

[B29] Malinda K. M. (2009). In vivo matrigel migration and angiogenesis assay. *Methods Mol. Biol.* 467 287–294. 10.1007/978-1-59745-241-0_17 19301678

[B30] Mao Q. Liang X. L. Zhang C. L. Pang Y. H. Lu Y. X. (2019). LncRNA KLF3-AS1 in human mesenchymal stem cell-derived exosomes ameliorates pyroptosis of cardiomyocytes and myocardial infarction through miR-138-5p/Sirt1 axis. *Stem Cell Res. Ther.* 10:393. 10.1186/s13287-019-1522-4 31847890 PMC6918658

[B31] Mathew S. A. Naik C. Cahill P. A. Bhonde R. R. (2020). Placental mesenchymal stromal cells as an alternative tool for therapeutic angiogenesis. *Cell. Mol. Life Sci.* 77 253–265. 10.1007/s00018-019-03268-1 31468060 PMC11104823

[B32] Nazari-Shafti T. Z. Neuber S. Garcia Duran A. Xu Z. Beltsios E. Seifert M. (2020). Human mesenchymal stromal cells and derived extracellular vesicles: translational strategies to increase their proangiogenic potential for the treatment of cardiovascular disease. *Stem Cells Transl. Med.* 9 1558–1569. 10.1002/sctm.19-0432 32761804 PMC7695640

[B33] Ning W. Li S. Yang W. Yang B. Xin C. Ping X. (2021). Blocking exosomal miRNA-153-3p derived from bone marrow mesenchymal stem cells ameliorates hypoxia-induced myocardial and microvascular damage by targeting the ANGPT1-mediated VEGF/PI3k/Akt/eNOS pathway. *Cell. Signal.* 77:109812. 10.1016/j.cellsig.2020.109812 33164880

[B34] Ou H. Teng H. Qin Y. Luo X. Yang P. Zhang W. (2020). Extracellular vesicles derived from microRNA-150-5p-overexpressing mesenchymal stem cells protect rat hearts against ischemia/reperfusion. *Aging (Albany. NY)* 12, 12669–12683. 10.18632/aging.102792 32657760 PMC7377831

[B35] Park H. Park H. Mun D. Kang J. Kim H. Kim M. (2018). Extracellular vesicles derived from hypoxic human mesenchymal stem cells attenuate GSK3β expression via miRNA-26a in an ischemia-reperfusion injury model. *Yonsei Med. J.* 59 736–745. 10.3349/ymj.2018.59.6.736 29978610 PMC6037597

[B36] Peng Y. Zhao J.-L. Peng Z.-Y. Xu W.-F. Yu G.-L. (2020). Exosomal miR-25-3p from mesenchymal stem cells alleviates myocardial infarction by targeting pro-apoptotic proteins and EZH2. *Cell Death Dis.* 11:317. 10.1038/s41419-020-2545-6 32371945 PMC7200668

[B37] Rani S. Ryan A. E. Griffin M. D. Ritter T. (2015). Mesenchymal stem cell-derived extracellular vesicles: toward cell-free therapeutic applications. *Mol. Ther.* 23 812–823. 10.1038/mt.2015.44 25868399 PMC4427881

[B38] Raso A. Dirkx E. Philippen L. E. Fernandez-Celis A. De Majo F. Sampaio-Pinto V. (2019). Therapeutic delivery of miR-148a suppresses ventricular dilation in heart failure. *Mol. Ther.* 27 584–599. 10.1016/j.ymthe.2018.11.011 30559069 PMC6403487

[B39] Shao C. Wang J. Tian J. Tang Y. D. (2020). Coronary artery disease: from mechanism to clinical practice. *Adv. Exp. Med. Biol.* 1177:2517. 10.1007/978-981-15-2517-9_1 32246442

[B40] Shinde A. V. Frangogiannis N. G. (2014). Fibroblasts in myocardial infarction: a role in inflammation and repair. *J. Mol. Cell. Cardiol.* 70 74–82. 10.1016/j.yjmcc.2013.11.015 24321195 PMC3995820

[B41] Takov K. He Z. Johnston H. E. Timms J. F. Guillot P. V. Yellon D. M. (2020). Small extracellular vesicles secreted from human amniotic fluid mesenchymal stromal cells possess cardioprotective and promigratory potential. *Basic Res. Cardiol.* 115:26. 10.1007/s00395-020-0785-3 32146560 PMC7060967

[B42] Teng X. Chen L. Chen W. Yang J. Yang Z. Shen Z. (2015). Mesenchymal stem cell-derived exosomes improve the microenvironment of infarcted myocardium contributing to angiogenesis and anti-inflammation. *Cell. Physiol. Biochem.* 37 2415–2424. 10.1159/000438594 26646808

[B43] Wang N. Chen C. Yang D. Liao Q. Luo H. Wang X. (2017). Mesenchymal stem cells-derived extracellular vesicles, via miR-210, improve infarcted cardiac function by promotion of angiogenesis. *Biochim. Biophys. Acta Mol. Basis Dis.* 1863 2085–2092. 10.1016/j.bbadis.2017.02.023 28249798

[B44] Wen Z. Mai Z. Zhu X. Wu T. Chen Y. Geng D. (2020). Mesenchymal stem cell-derived exosomes ameliorate cardiomyocyte apoptosis in hypoxic conditions through microRNA144 by targeting the PTEN/AKT pathway. *Stem Cell Res. Ther.* 11:36. 10.1186/s13287-020-1563-8 31973741 PMC6979357

[B45] Wu N. Zhang X. Du S. Chen D. Che R. (2018). Upregulation of miR-335 ameliorates myocardial ischemia reperfusion injury via targeting hypoxia inducible factor 1-alpha subunit inhibitor. *Am. J. Transl. Res.* 10, 4082–4094. 30662652 PMC6325516

[B46] Wu Q. Wang J. Tan W. L. W. Jiang Y. Wang S. Li Q. (2020). Extracellular vesicles from human embryonic stem cell-derived cardiovascular progenitor cells promote cardiac infarct healing through reducing cardiomyocyte death and promoting angiogenesis. *Cell Death Dis.* 11:2508. 10.1038/s41419-020-2508-y 32393784 PMC7214429

[B47] Xu H. Wang Z. Liu L. Zhang B. Li B. (2020). Exosomes derived from adipose tissue, bone marrow, and umbilical cord blood for cardioprotection after myocardial infarction. *J. Cell. Biochem.* 121 2089–2102. 10.1002/jcb.27399 31736169

[B48] Zhang C. Wang H. Chan G. C. F. Zhou Y. Lai X. Lian M. (2020). Extracellular vesicles derived from human umbilical cord mesenchymal stromal cells protect cardiac cells against hypoxia/reoxygenation injury by inhibiting endoplasmic reticulum stress via activation of the PI3K/Akt pathway. *Cell Transplant.* 29. 10.1177/0963689720945677 32864999 PMC7563023

[B49] Zhang L.-L. Xiong Y.-Y. Yang Y.-J. (2021). The vital roles of mesenchymal stem cells and the derived extracellular vesicles in promoting angiogenesis after acute myocardial infarction. *Stem Cells Dev.* 30 561–577. 10.1089/scd.2021.0006 33752473

[B50] Zhang Y. Huang R. Zhou W. Zhao Q. Lü Z. (2017). miR-192-5p mediates hypoxia/reoxygenation-induced apoptosis in H9c2 cardiomyocytes via targeting of FABP3. *J. Biochem. Mol. Toxicol.* 31:e21873. 10.1002/jbt.21873 27780314

[B51] Zhao J. Li X. Hu J. Chen F. Qiao S. Sun X. (2019). Mesenchymal stromal cell-derived exosomes attenuate myocardial ischaemia-reperfusion injury through miR-182-regulated macrophage polarization. *Cardiovasc. Res.* 115 1205–1216. 10.1093/cvr/cvz040 30753344 PMC6529919

[B52] Zhao Y. Sun X. Cao W. Ma J. Sun L. Qian H. (2015). Exosomes derived from human umbilical cord mesenchymal stem cells relieve acute myocardial ischemic injury. *Stem Cells Int.* 2015:761643. 10.1155/2015/761643 26106430 PMC4461782

[B53] Zhu J. Lu K. Zhang N. Zhao Y. Ma Q. Shen J. (2018). Myocardial reparative functions of exosomes from mesenchymal stem cells are enhanced by hypoxia treatment of the cells via transferring microRNA-210 in an nSMase2-dependent way. *Artif Cells Nanomed. Biotechnol.* 46 1659–1670. 10.1080/21691401.2017.1388249 29141446 PMC5955787

[B54] Zilun W. Shuaihua Q. Jinxuan Z. Yihai L. Qiaoling L. Zhonghai W. (2019). miRNA-181a over-expression in mesenchymal stem cell-derived exosomes influenced inflammatory response after myocardial ischemia-reperfusion injury. *Life Sci.* 256:118045. 10.1016/j.lfs.2019.116632 31278944

