# Supplementary figures and images for: miR-4732-3p in Extracellular Vesicles From Mesenchymal Stromal Cells Is Cardioprotective During Myocardial Ischemia
Source: Front Cell Dev Biol. 2021 Aug 31;9:734143. doi: 10.3389/fcell.2021.734143 (PMC8439391; doi:10.3389/fcell.2021.734143)

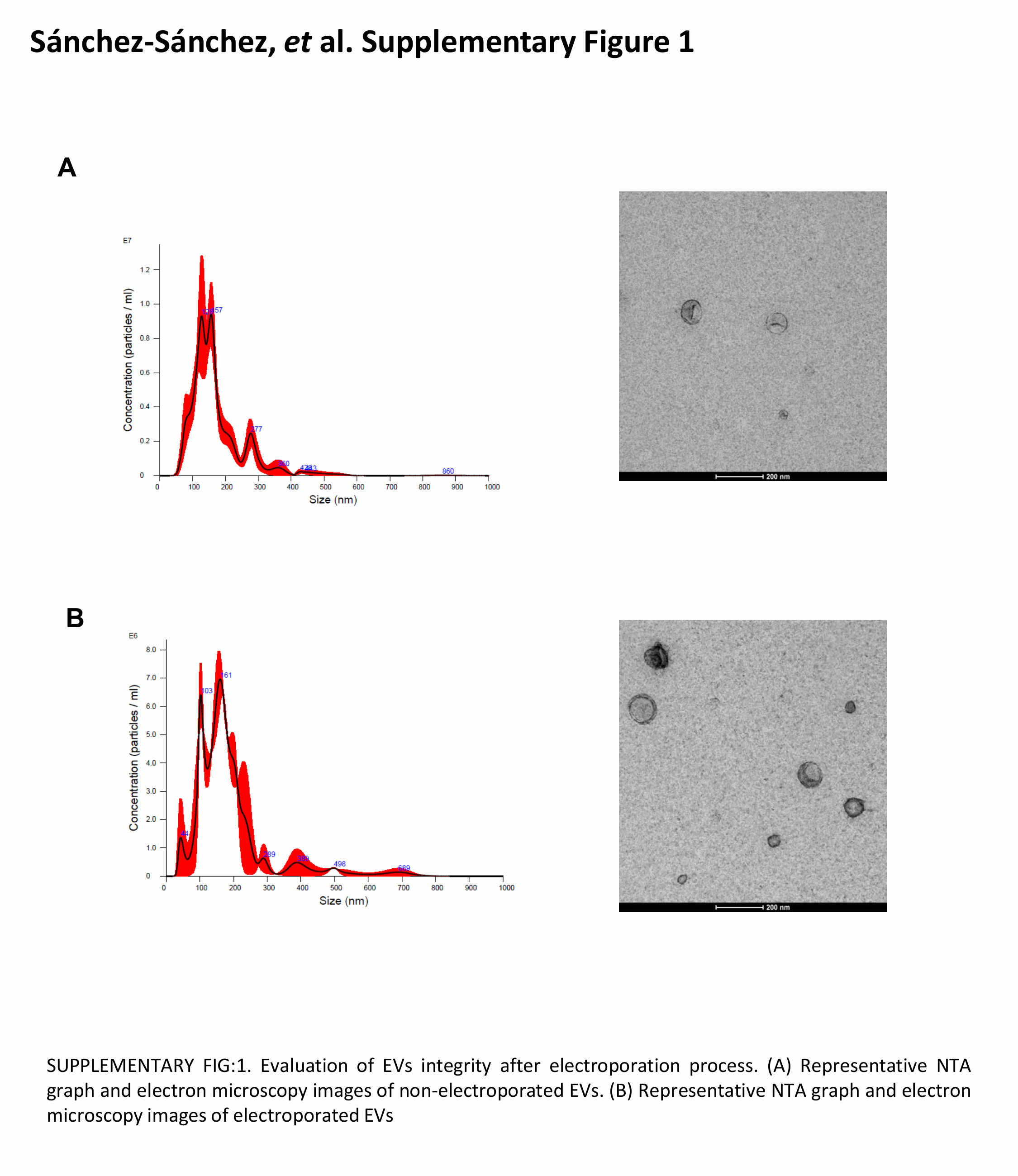

Supplement: Supplementary file 1 [file Image_1.TIF]

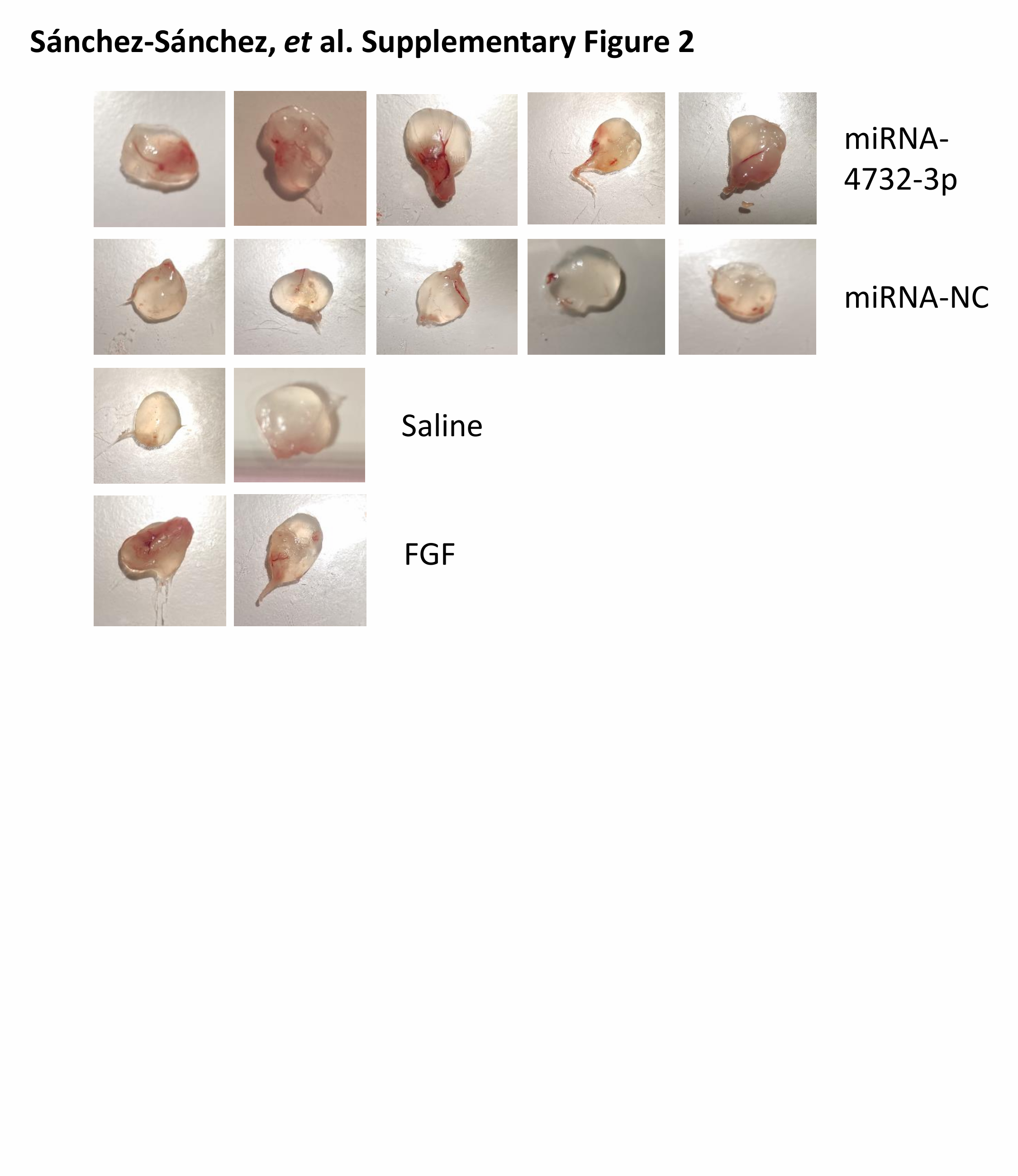

Supplement: Supplementary file 2 [file Image_2.TIF]
